# Considering interactive effects in the identification of influential regions with extremely rare variants via fixed bin approach

**DOI:** 10.1186/1753-6561-8-S1-S7

**Published:** 2014-06-17

**Authors:** Michael Agne, Chien-Hsun Huang, Inchi Hu, Haitian Wang, Tian Zheng, Shaw-Hwa Lo

**Affiliations:** 1 Department of Statistics, Columbia University, 1255 Amsterdam Avenue, Room 1005, MC 4690, New York, New York 10027, USA; 2 Department of Information Systems, Business Statistics and Operations Management, Hong Kong University of Science and Technology Business School, Hong Kong; 3 Division of Biostatistics, School of Public Health and Primary Care, the Chinese University of Hong Kong, Hong Kong

## Abstract

In this study, we analyze the Genetic Analysis Workshop 18 (GAW18) data to identify regions of single-nucleotide polymorphisms (SNPs), which significantly influence hypertension status among individuals. We have studied the marginal impact of these regions on disease status in the past, but we extend the method to deal with environmental factors present in data collected over several exam periods. We consider the respective interactions between such traits as smoking status and age with the genetic information and hope to augment those genetic regions deemed influential marginally with those that contribute via an interactive effect. In particular, we focus only on rare variants and apply a procedure to combine signal among rare variants in a number of "fixed bins" along the chromosome. We extend the procedure in Agne *et al *[1] to incorporate environmental factors by dichotomizing subjects via traits such as smoking status and age, running the marginal procedure among each respective category (i.e., smokers or nonsmokers), and then combining their scores into a score for interaction. To avoid overlap of subjects, we examine each exam period individually. Out of a possible 629 fixed-bin regions in chromosome 3, we observe that 11 show up in multiple exam periods for gene-smoking score. Fifteen regions exhibit significance for multiple exam periods for gene-age score, with 4 regions deemed significant for all 3 exam periods. The procedure pinpoints SNPs in 8 "answer" genes, with 5 of these showing up as significant in multiple testing schemes (Gene-Smoking, Gene-Age for Exams 1, 2, and 3).

## Background

**Figure 1:**
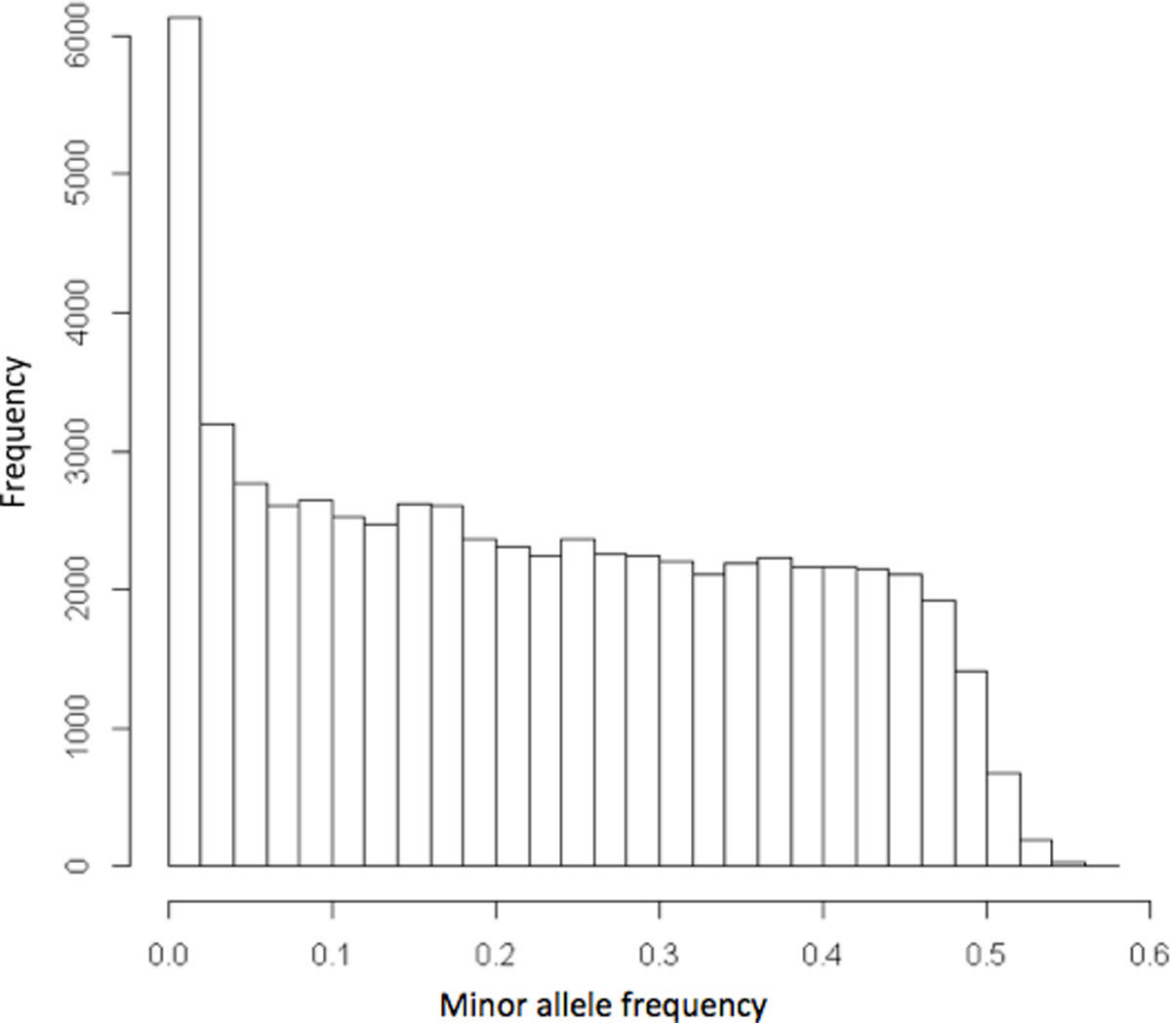
****Histogram of distribution of minor allele frequency in genotypes****.

The possible influence of rare variants on disease susceptibility has garnered more attention in recent years[2,3]. A rare variant is defined by a frequency of less than 1% [4]. Recent works have suggested that "uncommon or rare genetic variants can easily create synthetic associations that are credited to common variants" and have called for follow-up in future GWAS studies [5]. This field of "rare variants" will be the focus of this project. Figure 1 shows how skewed the distribution of minor allele frequency in the data is towards rare variants.

Hypertension as a disease has often been linked to environmental factors such as smoking status [6] and age [7]. In the past, methods of studying rare variants have been applied that deal only with a marginal genetic effect [1]. It would seem logical to attempt to incorporate some sort of interaction into the analysis of this study. In the past, "extreme groups analysis" (EGA) has been suggested as a method for dealing with an interaction between traits in a simple manner[8]. This method has both been praised and come under scrutiny for its simplicity and its tendency to only a more distilled version of the data [9].

Unfortunately, even those procedures that deal with rare variants [1-3] do not effectively address the issue of the potential interactive effect between environment and gene effects. Any simple procedure that attempts to address these gene-environment interactions, while simultaneously demonstrating a reasonable power to detect rare variants, would be quite ground breaking and novel.

One method of addressing "finding the useful information from the vast amounts of messy and noisy data available" builds on the method of partitions [10]. Among the claims of such a method is that "it has the advantage of avoiding a difficult direct analysis, involving possibly thousands of variables, by dealing with many randomly selected small subsets from which smaller subsets are selected, guided by a measure of influence" [10]. Such methods have been further developed to address interactions, showing that the "classification error rates can be significantly reduced by considering interactions" [11].

It should be noted that the methods proposed were developed and implemented without prior knowledge of the true causal single-nucleotide polymorphisms (SNPs) or the model generation process. Even though, upon learning of the true model, the attempt to identify interactive effects in the simulated data is doomed to be fruitless, the analysis is carried out in order provide a unique and repeatable procedure that can be applied to a more general data set. This is especially useful to consider real data has that have proved be full of interactions [6,7].

## Methods

### Data set

Genetic Analysis Workshop 18 (GAW18) contains whole genome sequencing (WGS) data in a pedigree-based sample. Longitudinal phenotype data for hypertension and related traits includes sex, age, year of examination, systolic and diastolic blood pressure, use of antihypertensive medications, and smoking status at up to 4 time points. A total of 200 replicates of simulated longitudinal phenotype data are provided based on real genotypes, pedigree structures, and trait distributions. Analysis in this study is focused on chromosome 3, which after eliminating with those SNPs without reference sequence contains two files of 773,088 and 62,915 SNPs, with 849 subjects with information on both genotype and phenotype data. Among the unrelated individuals, 142 subjects can be definitely mapped to subject IDs. We use the first replicate of the simulated data for our analysis.

### Fixed bins

In this study, we look only at chromosome 3. As in Agne *et al *[1], we implement a grouping of SNPs by bins of a fixed size (number of SNPs) to aggregate potential signal among rare variants. These non-overlapping bins span the entire chromosome. The use of fixed bins (whose size can be easily adapted) allows a computationally simple and flexible method for allocation of SNPs to regions; bypasses the need for additional gene or pathway information; and takes advantage of any meaningful associations based on genetic proximity on the chromosomes that might arise, such as via linkage disequilibrium. Most important, the collapsing of signal allows influential rare variants to be pinpointed, some of which might occur on genes that have very few or even a single SNP, when identification of such would be difficult because of their weak individual signals when the size of the region analyzed was a single SNP or a single gene. In Agne *et al *[1], the sensitivity to the size of bin is discussed, and it shown that such methods appear robust to any chose of bin size ranging from 30 to 100 SNPs. The choice of a particular bin size within that acceptable range was thus guided by computational concerns.

### Identification of marginally influential regions with private variants

We examine each exam period separately, and similar to Agne *et al *[1], we consider *case *subjects to be those with hypertension status = 1 and *control *subjects to be those with hypertension status = 0. In Agne *et al *[1], the simple score was implemented as follows:

$$\begin{array}{c} p_{i}=\frac{\left(\#\text{of private variants in fixed bin i, case}\right)}{\left(\#\text{of SNPs of each fixed bin i}\right)}\\ \;\;where\;i=1,...,\#of\text{ fixed bins}\text{.} \end{array}$$

This method can indeed be applied to the data at hand, but we will suggest that the updated interactive score, in the absence of interactive effects, will extract similar information.

### Dichotomy of subject with respect to environmental traits

We only consider one-way interactions between a single environmental trait and genetic information. To incorporate a potential interaction between SNPs and longitudinal traits, we establish a simple dichotomy of subjects via these traits. We consider smoking status and age. For smoking status, we take advantage of the natural dichotomy of smokers and nonsmokers, labeling nonsmokers as "low" on smoking status and smokers as "high" on smoking status. For age, we pick the median age as a cutoff, establishing those younger than the median age within an exam period as "low" on age and those at least as old as the median age as "high" on age. The median was especially convenient because it ensured an equal number of subjects in both the "high" and the "low" partitions. In practice, it is very similar to a two-means clustering approach, which has been proven effective in the past [11]. Partitions of more than two (especially in the age category, where this would be easy to implement) were considered, but empirical results have shown that "robust detection of interaction in feature selection is much more important than avoiding information loss by using the original variables" and that, in fact, "the more categories used the worse classification error rates" [11].

### Identification of Influential regions via interaction with environmental traits

To establish a potential interactive influence on disease status (for each longitudinal trait), we must combine the information between the "high" and "low" groups. To do so, we establish the following for each longitudinal trait (smoking status and age):

$${\text{p}}_{\text{i}},\;low=\frac{\left(\#\text{of}\;\text{rare}\;\text{variant}\;\text{among}\;\text{fixed}\;\text{bin}\;\text{i},\;\text{case},\;\text{among}\;\prime \prime \text{low}\prime \prime \;\text{subjects}\right)}{\left(\#\text{of}\;\text{SNPs}\;\text{of}\;\text{fixed}\;\text{bin}\;\text{i},\;\text{among}\;\prime \prime \text{low}\prime \prime \;\text{subjects}\right)}$$

$${\text{p}}_{\text{i}},\;high=\frac{\left(\#\text{of}\;\text{rare}\;\text{variant}\;\text{among}\;\text{fixed}\;\text{bin}\;\text{i},\;\text{case},\;\text{among}\;\prime \prime \text{high}\prime \prime \;\text{subjects}\right)}{\left(\#\text{of}\;\text{SNPs}\;\text{of}\;\text{fixed}\;\text{bin}\;\text{i},\;\text{among}\;\prime \prime \text{high}\prime \prime \;\text{subjects}\right)}$$

$$p_{i,\;interact}=p_{i,low}+p_{i,high}\;where\;i=1,\ldots ,\#\;of\;fixed\;bins$$

Because we are only interested in rare variants, our measure calculates the proportion of rare variants that are considered case (diseased) out of the total (case or control) number of rare variants in a given bin. To do so, the numerator is the number of rare variants that are affected by disease (case) in a given bin. The denominator is the total number of rare variants either affected or unaffected by disease (case or control) in a given bin. This statistic is then calculated for each (of the two) partitions of the subjects and summed to give the final interaction statistic.

In this article, we define "rare" variants as either having only 1 (private) or 2 minor alleles in the sample. Although the previous marginal procedure used only private variants as rare, any cutoff can be used. A more inclusive cutoff means a larger sample size of so-called "rare" variants.

### Significance via permutation test for interaction score

We use the standard procedure as established in Agne *et al *[1]. Affected status is randomly permuted among SNPs from each fixed bin, and the permutated *p*-value for fixed bin *i *is defined as #{observed *p_i,interact _*< permuted *p_i,interact_*}/1000. This ensures that regions with an unusually high observed *p_i _*value will produce a low *p*-value, indicating significance.

### Possible concordance of results between exam periods

Our method relies on treating each exam period as a separate data set. This ensures that no subject is counted more than once in any analysis. However, it will be interesting to examine any possible agreements, discrepancies, or trends regarding our results for the several exam periods, each of which can be considered a cross-sectional analysis and none of which overlap.

### Comparison of results between gene-smoking and gene-age effects

We are also interested in subjects that show as influential cross-sectionally during a single exam period via both the gene-smoking and gene-age tests.

## Results

**Figure 2:**
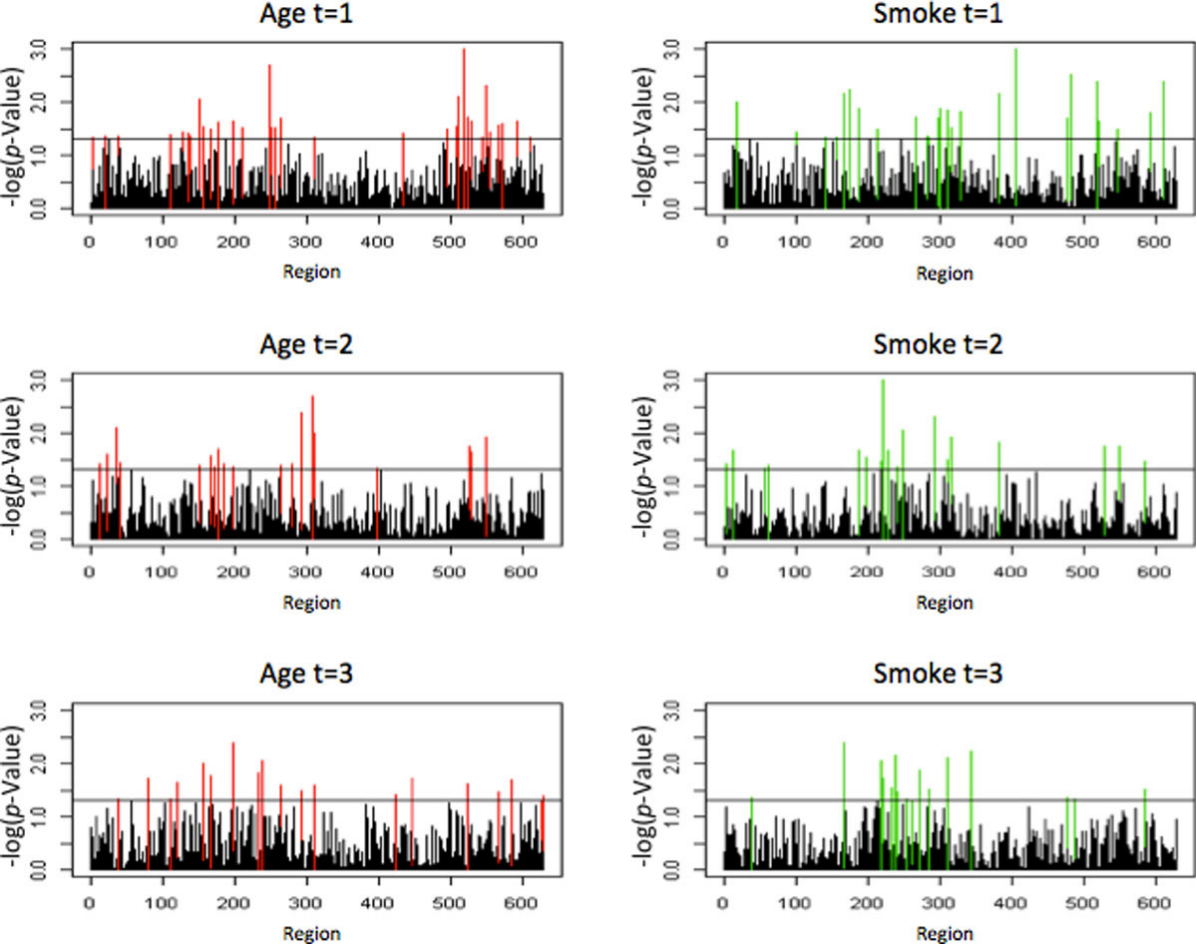
**Significance of regions for gene-smoking and gene-age analyses over 3 exam periods**.

We ran 1000 permutations investigating the interactive effect between rare variants and smoking status for the first, second, and third exam periods. We then ran 1000 permutations investigating the interactive effect between rare variants and age for each of those three time periods. The significance levels for each of 629 fixed-bin regions (of 100 SNPs each) is shown in Figure 2, with each of the 6 scenarios in a separate graph. We used 1 replicate in this analysis, but it can easily be extended to use all 200 replicates when doing so is economically and computationally feasible.

### Influential regions

We observe 58 instances of influential regions in the 3 exam periods via the gene-smoking analysis and 61 via the gene-age analysis. Among those, we observe SNPs in 8 "answer" genes, as displayed in Table 1.

**Table 1:** Answer single-nucleotide polymorphisms found

SNP	Gene	Significant tests
rs4681737	*ARF4*	GS-3
rs4681767	*ARHGEF3*	GA-1
rs1497266	*NMNAT3*	GA-1, GA-2, GS-1, GS-2
rs9843488	*PDCD6IP*	GA-1, GA-2
rs7625177	*PPP2R3A*	GA-1, GS-1
rs2526397	*SEMA3F*	GS-2, GS-3
rs13323368	*SUMF1*	GS-2, GS-3
rs9877046	*TUSC2*	GS-3

For example, GS-1 refers to gene-smoking, exam period 1.

*SNP*, single-nucleotide polymorphism.

### Overlapping regions between exam periods and methods

There were multiple overlapping regions for exam periods via the gene-smoking analysis (11 totals). There were also multiple overlapping regions for exam periods via the gene-age analysis (15 total, with 4 overlapping all three exam periods). There were also several overlapping regions between the gene-smoking and gene-age analyses (6 for exam period 1, 5 for exam period 2, and 8 for exam period 3). Among these, 5 instances of SNPs in "answer" genes showing up as significant in more than 1 testing period and/or interaction scheme (gene-age or gene-smoking) were observed. These are shown in Table 1. For example, SNPs in the gene *NMNAT3 *were highlighted as influential in the both the gene-smoking and gene-age analyses for both exam periods 1 and 2.

### Analysis of type I error and power

Ultimately, the goal of any such method would be to evaluate the power of a statistic in the context of its false-positive rate. However, because true interactions were not actually simulated in the data set, power analysis would not be appropriate. Although there is the potential for the method to pick up signal from marginal effects, the previous work [1] with fixed bins more directly addresses this issue. With regard to the false-positive rate, this method is a screening method whose goal is to reduce the vast number of potential SNPs for a biologist to analyze down to a more manageable number. Although within each fixed bin, the method provides a bin-wise significance level of 0.05 from permutation tests, we are not concerned about correcting for the multiple comparisons of a chromosome-wide significance because we simply wish to screen SNPs as potential "candidates" as opposed to definitely calling them influential.

## Discussion

Although analysis involving the influence of common variants is well established, methods tackling the issue of influential rare variants are still needed. The method outlined here will have little power to determine influential common variants, but as a result, its potential to pinpoint extremely rare variants of an influential nature will be very difficult to match by a common variant approach. Moreover, this method goes beyond simple marginal methods and has the potential to identify influential genetic regions that might only exhibit a strong association with disease when considering interaction with other dynamic traits such as smoking status and age.

The method does, of course, have limitations. For instance, if both effects that are positively and negatively (protective) associated with disease are present in a given bin, the effects may cancel. This is one disadvantage of this statistic over the similar *I score *statistic as proposed in [10,11], which contains a symmetric square function. However, the method proposed in this paper only needs have effects that go in the same direction within a given fixed bin. If there are causal effects in one gene and protective effects in another, the procedure could detect both. The analogous two-sided test statistic would be used for this purpose. Because the influential SNPs are relatively sparse, the chance of both directions of effects appearing in the same bin seems small. This might also be a motivation to use a bin size on the smaller size (as small as 30 SNPs wide).

Deeper interactions, such as those between smoking and age, might be examined. Furthermore, the extension to multiple replicates is easy by the ranking method of return frequency as outlined in Agne *et al *[1]. We note, however, that the benefits of using multiple replicates can be sometimes far outweighed by the cost of acquiring and analyzing these new subjects, especially in real-world examples.

## Competing interests

The authors declare that they have no competing interests.

## Authors' contributions

SHL, MA, and CHH designed the study. MA, CHH, SHL, and TZ performed the study. MA, CHH, IH, HW contributed to analysis of the data. MA, CHH, and SHL drafted the manuscript. All authors read and approved the final manuscript.
